# Using anti-Müllerian hormone to predict premature ovarian insufficiency: a retrospective cross-sectional study

**DOI:** 10.3389/fendo.2024.1454802

**Published:** 2024-11-19

**Authors:** Yuanxin Huang, Xiaojun Kuang, Huiting Jiangzhou, Meiling Li, Dongjian Yang, Dongmei Lai

**Affiliations:** ^1^ The International Peace Maternity and Child Health Hospital, School of Medicine, Shanghai Jiao Tong University, Shanghai, China; ^2^ Guangdong Province Women and Children Hospital, Guangzhou, China; ^3^ Shanghai Key Laboratory of Embryo Original Diseases, Shanghai, China

**Keywords:** premature ovarian insufficiency (POI), anti-Mullerian hormone (AMH), follicle stimulating hormone (FSH), big data, reproductive health

## Abstract

**Background:**

Premature ovarian insufficiency/failure (POI/POF) is a significant issue for women of reproductive age. Anti-Müllerian hormone (AMH) is a potential biomarker of ovarian reserve, but its clinical value in diagnosing and predicting POI/POF remains unclear. This study aimed to analyze the correlation between AMH and basal follicle-stimulating hormone (FSH) levels in women aged 18 to 40 and evaluate AMH’s predictive value for POI/POF.

**Methods:**

A total of 21,143 participants aged 18-40 who visited the gynecology department or underwent physical examinations at the International Peace Maternity and Child Health Hospital in Shanghai, China, from July 2016 to June 2021 were enrolled. Demographic information and laboratory test results were collected, including age, FSH, AMH, E2 and test dates. Participants were grouped by FSH and AMH levels, and subgroup analyses were performed to investigate the relationship between these hormones and age. The AMH level associated with POI risk was evaluated using restricted cubic splines (RCS) and logistic regression. Clinical benefit was assessed by decision curve analysis (DCA).

**Results:**

Participants with higher FSH levels had significantly lower median AMH levels and vice versa(p<0.001). At AMH ≥ 0.5 ng/mL, FSH levels were normal or slightly elevated with age. At AMH level below 0.5ng/ml,basal FSH increased significantly with age. At FSH <10 IU/L, AMH levels show a trend of rising and then decreasing with age, reaching a peak at approximately 25 years old and gradually decreasing with age. At FSH ≥10 IU/L, AMH levels show a gradual downward trend with age, and at FSH >40 IU/L, AMH levels remain very low to undetectable values. The RCS showed that the risk of POI/POF in the overall population sharply increased until serum AMH reached a low level (below 0.5ng/ml). DCA showed that a low AMH level had good clinical diagnostic utility in predicting POI/POF.

**Conclusion:**

Our analysis of a large dataset suggests that serum AMH levels are inversely correlated with FSH levels and that AMH is a good predictor of POI until it drops to a low level.

## Introduction

Premature ovarian insufficiency (POI) is a complex and refractory disorder. It manifests as irregular menstruation or early menopause, with elevated serum follicle-stimulating hormone (FSH) and reduced estradiol (E2) levels in women under 40. The diagnostic criteria depends on elevated FSH levels (>25 IU/L) on two occasions over 4 weeks apart (1, 2). When FSH levels exceed 40 IU/L on two occasions, it’s classified as premature ovarian failure (POF) (1), representing an advanced stage of POI progression, leading to not only infertility but also long-term squeal complications, such as cardiovascular disease, osteoporosis, and neurocognitive disorders, even premature death. Estrogen deprivation and cardiovascular disease risk in primary ovarian insufficiency. POI/POF is heterogeneous in etiology, and known causes include genetic, autoimmune, iatrogenic or idiopathic factors (3). MRKH syndrome, the absence or underdevelopment of the uterus and upper part of the vagina can affect the development and function of the ovaries, potentially leading to ovarian insufficiency (4).

Ovarian dysfunction is associated with a reduction in both the quantity and quality of oocytes. As women age, their fertility naturally declines, an occurrence associated with a reduction in the quantity of primordial follicular reserve, a condition known as diminished ovarian reserve (DOR).

The basal serum levels of basal follicle-stimulating hormone (*FSH*), luteinizing hormone (LH), estradiol (E2), and *FSH*: LH ratio, inhibit B, anti-Müllerian hormone (AMH) and antral follicle count (AFC), which have been proven to be predictors of oocyte quantity (5). FSH is the sole indicator employed for the diagnosis of premature ovarian insufficiency (POI), but its usefulness is limited due to its high variability between or within menstrual cycles (6). Moreover, many patients may experience a stage when FSH is normal or slightly increased before being diagnosed with POI, which increases the complexity of the clinical identification of POI. The anti-Müllerian hormone (AMH) in the serum of women of reproductive age is secreted by the granulosa cells of ovarian follicles and appears to regulate early follicle development. Its level varies slightly with the menstrual cycle, reaching the peak value during the late follicular phase (7). AMH and AFC have recently been considered more promising for assessing ovarian reserve, given their high sensitivity and specificity in predicting ovarian response and good intracycle reliability (8).

Currently, prior research has primarily enrolled healthy women or women with specific medical conditions, resulting in a dearth of data from a sizable population (9, 10). The extent to which AMH can serve as the most valuable indicator of ovarian dysfunction remains to be substantiated through further investigation. Furthermore, the hormonal fluctuations associated with ovarian dysfunction are intricate and constantly changing. Previous studies have solely employed linear regression analysis, which lacks comprehensive and robust evidence, thereby constraining its clinical applicability. The objective of this study was to elucidate the intricate association between serum levels of AMH and ovarian dysfunction in a more comprehensive manner.

## Materials and methods

### Participants and protocols

This was a retrospective cross-sectional study of women who came to the gynecology department or underwent a physical examination at the International Peace Maternity and Child Health Hospital in Shanghai, China, from July 2016 to June 2021. The study protocol was approved by the Medical Ethics Committee of the International Peace Maternity and Child Health Hospital in Shanghai, China. Patients ‘clinical information was recorded by specialists of gynecology in the hospital information system. The inclusion criteria were as follows: (1) AMH and sex hormone tests obtained at the same time in the early follicular phase to ensure consistency and minimize variability due to the menstrual cycle; (2) age between18-40 years; (3) no history of radiotherapy or chemotherapy; (4) no history of hysterectomy, oophorectomy, or any other type of ovarian surgery; and (5) no ovarian tumors, PCOS, or history of metabolic diseases. Chromosomal analysis and FMR-1 gene premutation testing were not routinely performed in all patients, and therefore, these test results were not considered in the scope of this study. After screening, 21,143 outpatients were included.

Participants’ age and hormonal levels (FSH, luteinizing hormone (LH), AMH, and E2) were recorded. FSH, LH, and E2 were determined via chemiluminescence assay (Beckman Coulter DXI800, USA). AMH was determined via a chemiluminescence assay (Kangrun, China). AMH levels below 0.06 ng/mL (limit of detection, LoD) were assigned a randomized value between 0-0.06 ng/ml during the statistical process.

### Statistical analysis

Data management was performed using Microsoft SQL Server (Microsoft Corp), and all statistical analyses were conducted using R software version 4.1.2 (Institute for Statistics and Mathematics, Vienna, Austria; https://www.r-project.org/). The normal distribution of quantitative variables was assessed using the Kolmogorov–Smirnov test. Continuous variables are reported as medians and interquartile ranges (IQR), while categorical variables are reported as whole numbers and percentages. In the descriptive analysis, the associations between patients’ demographic characteristics and laboratory tests were assessed. Subgroup analysis was performed using stratification by age, FSH level, and AMH level. The age groups were 18≤age<25, 25≤age<30, 30≤ age <35, and 35≤ age <40 years. According to the diagnostic criteria outlined in the ESHRE guideline for the management of women with premature ovarian insufficiency, POF is defined as amenorrhea with elevated FSH levels (>40 IU/L), while POI is characterized by elevated FSH levels (>25 IU/L) on two separate occasions at least four weeks apart in women under 40 years of age. Patients were accordingly subdivided into four groups:(FSH<10 IU/L(normal),10≤FSH ≤25 IU/L(elevated FSH),25<FSH ≤ 40 IU/L(POI), and FSH>40 IU/L (POF) (1). The reference range for Anti-Müllerian Hormone (AMH) varies with age, and currently, there is no established specific AMH threshold for diminished ovarian reserve. However, several consensus guidelines do refer to an AMH range of 0.5-1.1 ng/mL, as suggested by the Bologna Criteria, as an indicator of diminished ovarian function. Patients were accordingly subdivided into three groups: AMH ≥1.1ng/ml, 0.5≤AMH<1.1ng/ml, AMH<0.5 ng/ml. The parameters of each group were subsequently calculated by scatter plot and nonlinear regression analysis with a 95% confidence interval (CI). Comparison of hormonal variables among subgroups of participants in the study was analyzed using the Kruskal-Wallis H-test.

Multivariable logistic regression analysis began with the following clinical candidate predictors: age, AMH, LH, and E2. Candidate predictors were applied to develop a diagnostic model for POI. Backward stepwise selection was applied by using the likelihood ratio test with Akaike’s information criterion as the stopping rule (11, 12). A forest plot was conducted to represent the results based on multivariable logistic analysis. In addition, we used the receiver operator characteristic (ROC) curve to estimate the diagnostic value of AMH and LH.

The associations between levels of AMH and the risk of POI were evaluated on a continuous scale with restricted cubic spline (RCS) curves based on logistic regression models with 95% confidence intervals. To balance best fit and overfitting in the main splines for POI, the number of knots, between three and seven, was chosen as the lowest value for the Akaike information criterion. Variable-adjusted statistical analyses were adjusted for age (as time scale).

Decision curve analysis (DCA) was conducted to determine the clinical usefulness of AMH by quantifying the net benefits at different threshold probabilities (13). The statistical significance level was considered at a p value <0.05.

## Results

### Clinical characteristics

**Figure 1:**
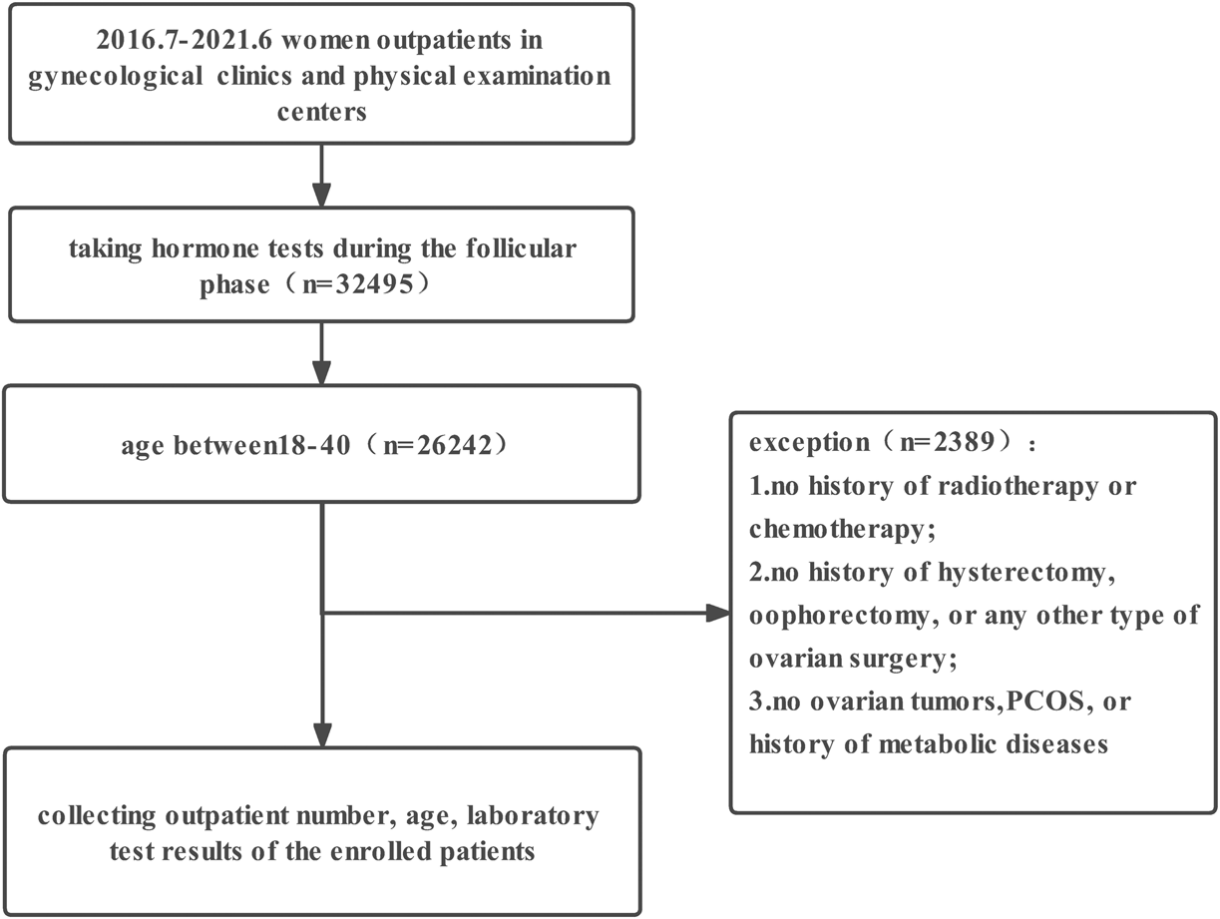
Study inclusion and exclusion flow chart. PCOS, Polycystic ovary syndrome.

A total of 21,143 participants who met the criteria were enrolled in our study (**Figure 1**), and they were evaluated for AMH, FSH, LH, and E2 levels in each age group (**Supplementary Table 1**). There were1,831 women aged 18-25(8.7%), 6,669 women aged 25-30(31.5%), 7,698 women aged 30-35 (36.4%), and 4,945 women aged 35-40(23.4%).The mean age of the participants was 31.66 years. The older groups had lower median AMH levels and higher median FSH levels (**Supplementary Table 1**, p<0.0001). According to FSH level, patients were divided into four groups: normal (n=16,538,78.2%), elevated FSH (n=4,043,19.1%), POI(n=300,1.4%), and POF(n=262,1.2%). Participants with higher FSH levels had significantly lower median AMH levels and higher median LH levels (**Supplementary Table 1**, p<0.001). Median E2 levels were higher in the group with higher FSH levels than in the group with lower FSH levels when FSH <40 IU/L, but were lowest in the POF group. Likewise, according to AMH level, patients were divided into three groups: AMH ≥1.1ng/ml (n=17,766,84.0%), 0.5≤AMH<1.1ng/ml, (n=1,845,8.7%), and AMH<0.5 ng/ml (n=1,532,7.3%). Participants with lower AMH levels had a significantly higher median FSH level (**Supplementary Table 1**, p<0.001). Evaluation of the levels of other hormones among the groups did not show any significant increasing or decreasing trends.

### AMH group analysis

**Figure 2:**
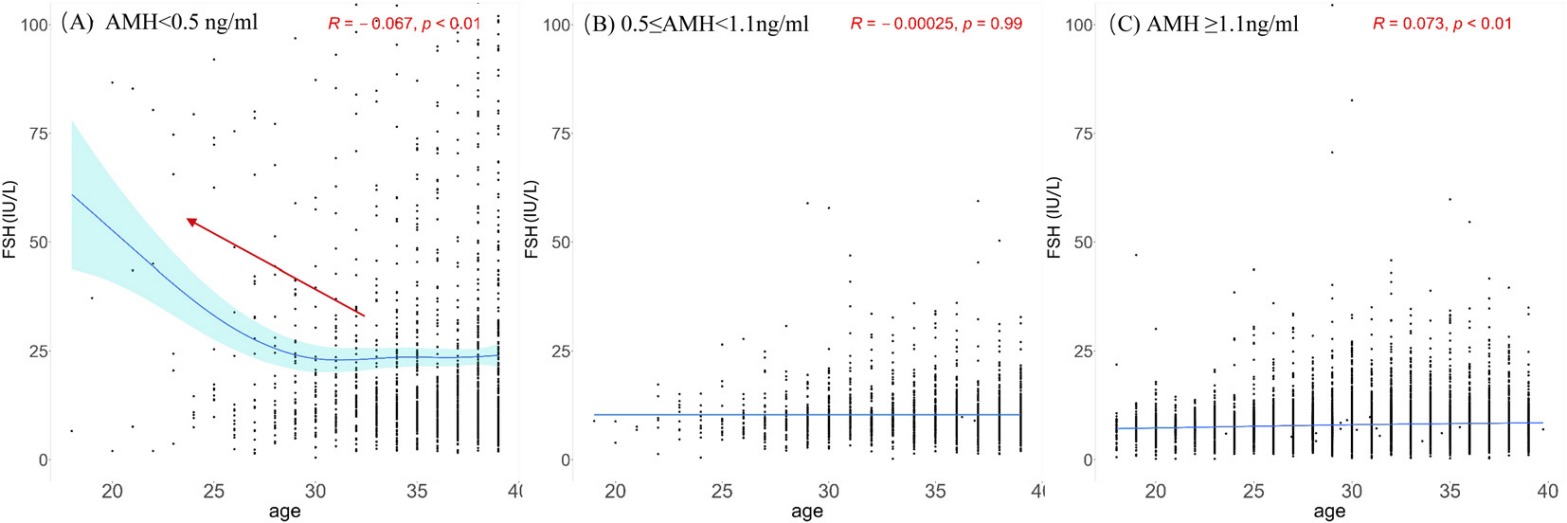
Scatterplots between FSH and age among different AMH groups. **(A)** AMH < 0.5 ng/ml; **(B)** 0.5 ≤ AMH< 1.1 ng/ml; **(C)** AMH ≥1.1ng/ml. The black dots illustrate the FSH levels of individuals across various ages. The blue solid line represents the trend line fitted through the data points, reflecting the overall trend of FSH levels with age. Light blue region represents the 95% confidence interval. Red arrow highlights the comparatively elevated FSH levels in younger people observed within group.

Scatter plots were plotted with age and FSH levels of the three groups divided by AMH level. Nonlinear fittings were performed, and 95% confidence intervals were calculated, as shown in **Figure 2**. When AMH was ≥0.5 ng/ml, the FSH level did not change significantly with age. When AMH was <0.5 ng/ml, the participants had higher FSH levels. Scatter plots were generated to examine the relationship between AMH levels and FSH levels. Nonlinear regression analyses were conducted, and 95% confidence intervals were computed, as depicted in **Supplementary Figure 1**. In cases where AMH levels were equal to or greater than 0.5 ng/ml, the basal FSH level exhibited relative stability or only a slight increase. Conversely, when AMH levels were less than 0.5 ng/ml, the basal FSH level displayed significant fluctuations within a wide range, demonstrating a clear upward trend as the AMH level decreased.

### FSH group analysis

**Figure 3:**
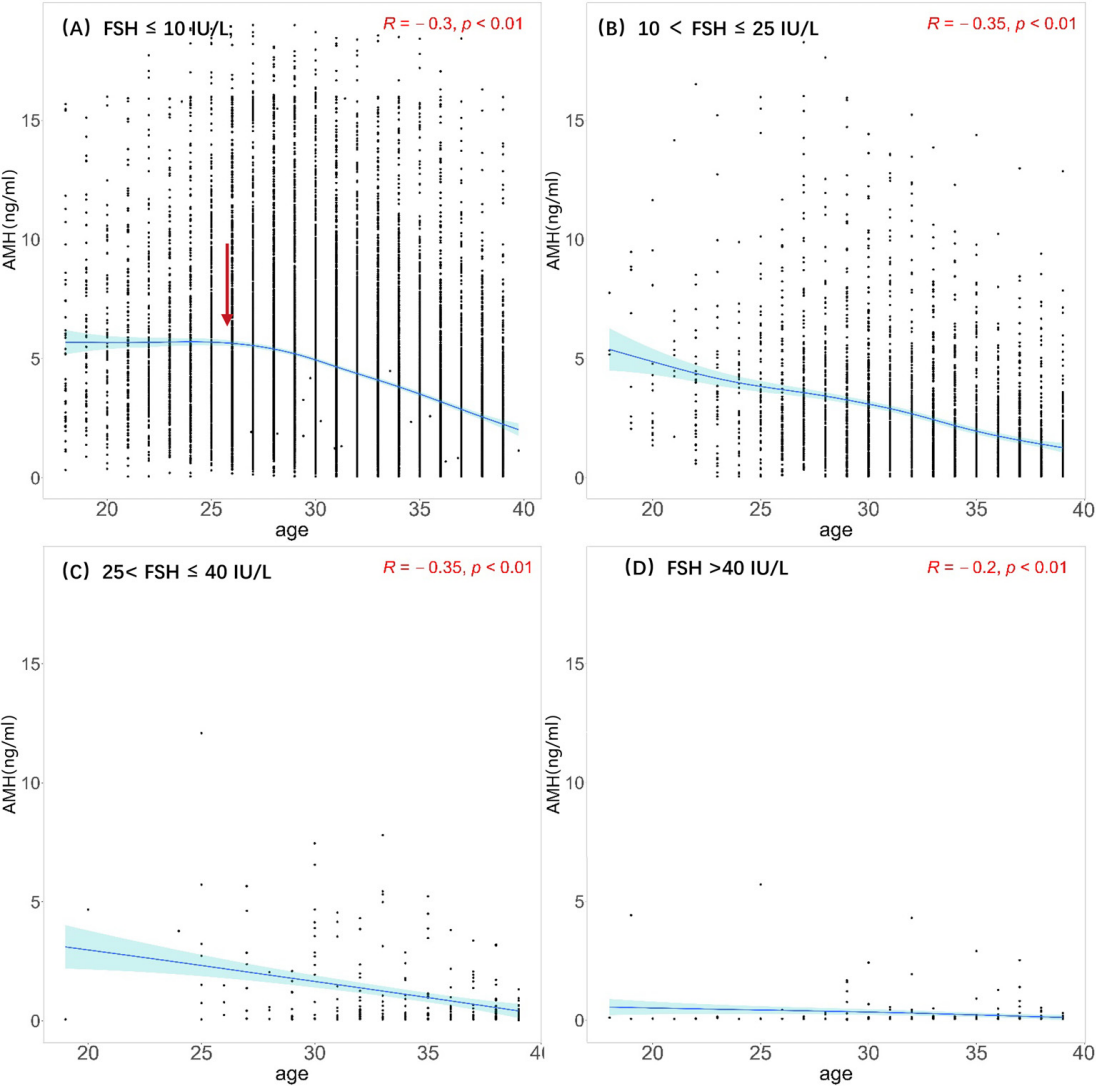
Scatterplots between AMH and age among different FSH groups. **(A)** Normal: FSH ≤ 10 IU/L; **(B)** elevated FSH:10 < FSH ≤ 25 IU/L; **(C)** POI: 25< FSH ≤ 40 IU/L; **(D)** POF: FSH >40 IU/L The black dots illustrate the AMH levels of individuals across various ages. The blue solid line represents the trend line fitted through the data points, reflecting the overall trend of AMH levels with age. Light blue region represents the 95% confidence interval. Red arrow represents that AMH levels reaching a peak at approximately 25 years old.

Scatter plots were generated with age and AMH levels of the four groups divided by FSH level. Nonlinear fittings were performed, and 95% confidence intervals were calculated, as shown in **Figure 3**. At FSH <10 IU/L (normal group), AMH levels show a trend of rising and then decreasing with age, reaching a peak at approximately 25 years old and gradually decreasing with age. At FSH ≥10 IU/L, AMH levels show a gradual downward trend with age, and at FSH >40 IU/L(POF group), AMH levels remain very low.

### POI prediction

**Figure 4:**
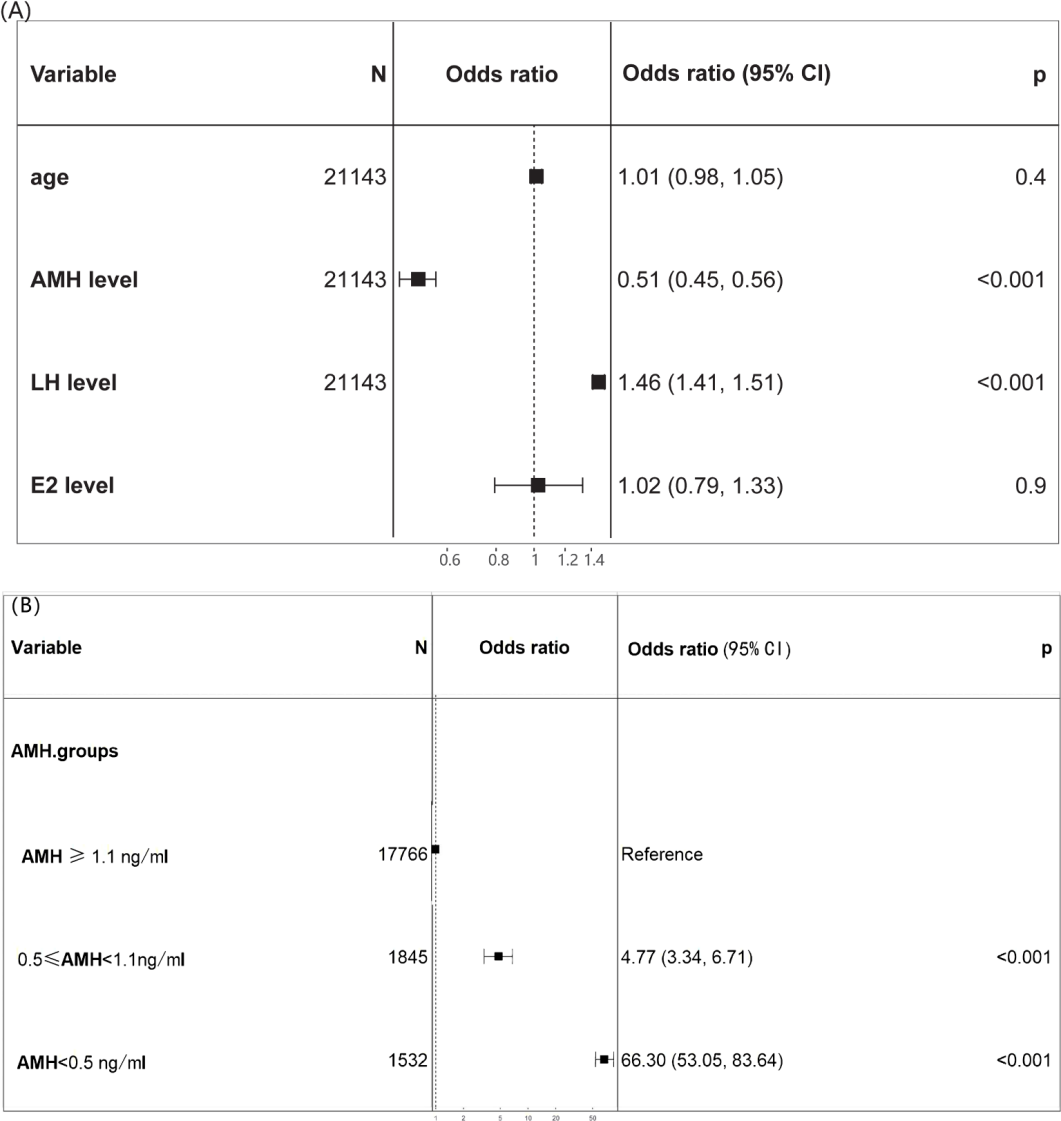
Forest plot of the diagnostic odds ratio. **(A)** The multivariate analysis identified age, AMH, LH, E2 as candidate predictors for POI; **(B)** odds ratio of various AMH levels for POI.

Preoperatively identifiable variables were included for logistic regression analysis to further predict the risk of POI. In multivariate analysis, AMH (odds ratio 0.36) and LH (odds ratio 1.45) were identified as risk factors for POI (P<0.001) (**Figure 4**), while age and E2 showed no significance. Compared to normal AMH levels, low AMH levels had strong effects on predicting POI: AMH < 0.5 ng/ml(odds ratio 66.3) and 0.5 ≤ AMH< 1.1 ng/ml(odds ratio 4.77). For predicting POI, AMH had the best predictive value (AUC 0.900, 95% CI: 0.883-0.918) for both sensitivity and specificity, while LH had a lower predictive value (AUC 0.865, 95% CI: 0.843-0.883) (**Supplementary Figure 2**).

The central position of the bar marks the point estimate of the effect size for each variable on POI diagnosis. Horizontal lines extending from the bars represent the confidence intervals for the effect sizes.

The relationship between AMH levels and the diagnosis of POI was estimated by the method of restricted cubic splines (**Supplementary Figure 3**). Low levels of AMH (AMH <0.5 ng/ml) were associated with a sharply increased risk of POI in the overall population. AMH appeared to have a significant association with the risk of POI, having a hazard ratio (HR) of approximately 1.0 (p value for interaction between AMH levels and risk of POI was <0.001).

The decision curve analysis for AMH is presented in **Supplementary Figure 4**. The decision curve showed that if the threshold probability is less than 22%, < using the AMH to predict POI is more beneficial than either the treat-all-patients scheme or the treat-none scheme. The intersection points of the treat-all-patients line and x-axis represent the prevalence of POI in the sample. Within this range, the net benefit was comparable, with several overlaps, based on AMH.

## Discussion

In this study, we investigated the correlations between AMH, FSH, LH, and E2 in women within the childbearing age range (18-40 years). Generally, there was a noticeable decline in AMH levels as age increased. However, the specific changes in AMH levels at different FSH levels varied. The process of ovarian aging is linked to a reduction in both the quantity and quality of ovarian primordial follicular pools. Previous researches have established a model illustrating age-specific alterations in AMH levels in premenopausal women, with a peak occurring at 24.5 years of age followed by a subsequent decrease until menopause (14). In accordance with this finding, a comparable pattern was observed solely when FSH levels were below 10 IU/L, with the peak of AMH levels occurring at approximately 25 years of age. Nonetheless, this phenomenon disappeared when FSH levels reached or exceeded 10 IU/L, as evidenced by a gradual decline in AMH levels with the growing age. Furthermore, when FSH levels exceeded 40 IU/L, the AMH levels were too low to be detected. Our study demonstrates a correlation between an elevation in basal FSH levels and a reduction in AMH levels among the participants.

Our study indicated that an elevation in basal FSH was accompanied by a decline in AMH levels among participants. This outcome aligns with previous research demonstrating a negative association between AMH and FSH (15). Specifically, the basal FSH level remained relatively stable or exhibited a slight increase as the AMH level decreased but experienced a sudden increase when the AMH level fell below the threshold of 0.5 ng/mL. Systemic FSH levels rise in response to the lack of negative feedback from inhibin B and gradual declines in progesterone and estradiol (16). Recent reports have shown that the development of luteal phase dominant follicles (LPDFs) during the menopausal transition is associated with reduced luteal function accompanied by higher estradiol, lower progesterone, and lower inhibin A (17). Clinically, we observed irregular menses in many patients who had normal FSH and abnormally elevated estradiol levels before being diagnosed with POI, which complicated the early identification of POI.

It was reported that there was a positive collinear relationship between age and FSH in fertile females (16). However, our results demonstrated that there was a positive correlation between age and FSH only when AMH ≥1.1ng/ml. When 0.5 ≤ AMH< 1.1 ng/ml, no significant correlation was found between age and FSH. Further, there was a negative correlation between age and FSH when AMH < 0.5 ng/ml. Therefore, FSH does not necessarily increase with age and still depends on ovarian reserve. However, in the case where there is a limited amount of data for women under 25 years old with AMH levels below 0.5 ng/ml, the high FSH (follicle-stimulating hormone) levels observed in some individuals may affect the accuracy of the results. These individuals are more likely to experience ovarian function decline due to genetic, autoimmune, environmental, or other disease factors. As predicted, the risk of developing POI increased with decreasing AMH levels. However, it was not a simple linear relationship. The risk of developing POI remained stable until AMH declined to a threshold. As AMH continued to decline, the risk of developing POI steeply increased. At present, except for basal FSH, no standardized reference or cutoff value is available for POI diagnosis. AMH and AFC have been considered with high hopes for assessing ovarian reserve, given their high sensitivity and specificity in predicting ovarian response and good intracycle reliability (18, 19). A recent study also suggested that AMH showed the highest predictive value for non-POI (AUC 0.932, 95% CI: 0.918-0.945) and POI (AUC 0.944, 95% CI: 0.933-0.954) (6). However, according to our study, caution should be taken when using AMH as a predictor of POI unless its level is low enough.

The risk-prediction performance, discrimination and calibration, could not capture the clinical consequences of a particular level of discrimination or degree of miscalibration (19–21). Therefore, to justify the clinical usefulness, we assessed whether it is valuable to use AMH to predict POI, and decision curve analysis was applied in this study. This method offers insight into clinical consequences based on threshold probability, from which the net benefit could be derived. Net benefit is defined as the proportion of true positives minus the proportion of false positives, weighted by the relative harm of false-positive and false-negative results (19). The decision curve showed that if the threshold probability of a patient was under 22%, using AMH obtained more benefit than either the treat-all-patients scheme or the treat-none scheme. When patients present with irregular menstrual symptoms, the diagnostic criterion for premature ovarian insufficiency (POI) only consider the assessment of follicle-stimulating hormone (FSH). In China, anti-Müllerian hormone (AMH) is currently tested at the patient’s expense, which can be slightly expensive. However, it is important to note that in some cases, only FSH level is checked, even if the patient’s AMH level is already significantly low. This approach may fail to attract attention and prompt timely intervention, despite the high probability of developing POI. In the context of POI, both AMH and FSH are valuable markers for assessing ovarian status and guiding treatment decisions. AMH provides a more stable and consistent measurement, while FSH helps confirm the diagnosis and assess the severity of ovarian insufficiency. Therefore, we recommend performing both AMH and FSH tests in clinical practice to provide a comprehensive evaluation of ovarian function in POI patients, aiding in the timely diagnosis and appropriate management of this condition.

The study’s strengths lie in its utilization of extensive datasets and thorough examination of the correlation between FSH and AMH, which substantiates the notion that AMH is a more sensitive indicator of ovarian dysfunction compared to FSH. However, due to the lack of complete information for all participants, multivariate analysis was not carried out to fully elucidate the possible factors affecting ovarian insufficiency. Although most patients in our study did not undergo just one blood test; they had multiple tests over time. However, for statistical purposes, we did not include all these tests in our analysis, as the large sample size could potentially mitigate individual variations due to the inherent randomness in AMH and FSH levels.

Patients diagnosed with POI may not have very regular menstrual cycles. Therefore, this study did not conduct specific statistics on this. We could estimate the average time interval based on their reported menstrual history. Further prospective longitudinal studies may be needed in the future to confirm the predictive role of different indicators of ovarian function. Furthermore, based on the current AMH detection methods, the AMH levels of many individuals are too low to obtain accurate numerical values, making it impossible to conduct detailed assessments to further predict the risk of POI. Thus, an ultrasensitive AMH detection method is urgently needed to provide a more accurate evaluation of decreased ovarian reserve.

## Data Availability

The raw data supporting the conclusions of this article will be made available by the authors, without undue reservation.
