# Supplementary material for: Using anti-Müllerian hormone to predict premature ovarian insufficiency: a retrospective cross-sectional study
Source: Front Endocrinol (Lausanne). 2024 Nov 19;15:1454802. doi: 10.3389/fendo.2024.1454802 (PMC11611575; doi:10.3389/fendo.2024.1454802)
Supplement: Supplementary file 1 [file DataSheet1.docx]

**Supplemental Online Content**

**The Relationship between Anti-Müllerian Hormone and Premature Ovarian Insufficiency: A Retrospective Cross-sectional Study**

Yuanxin Huang, M.D.,^a,b,c,#^ Xiaojun Kuang, M.D.,^a,c,#^ Huiting Jiangzhou, M.D.,^a,c^  Meiling Li, M.D.,^a,c^ Dongjian Yang, M.D.,^a,c^ Dongmei Lai, Ph.D.,^a,c,^*

**Table of contents**

Ⅰ. Supplemental table 1

Ⅱ. Supplemental figure 1

Ⅲ. Supplemental figure 2

Ⅳ. Supplemental figure 3

Ⅴ. Supplemental figure 4

| Characteristics | n | age | AMH (ng/mL) | FSH (IU/L)) | LH (IU/L) | E2 (pmol/L) |
| --- | --- | --- | --- | --- | --- | --- |
| Age groups | | | | | | |
| 18-25 | 1,831 | — | 4.61 [2.86, 7.16] | 7.30 [6.00, 8.90] | 4.10 [2.80, 5.50] | 140.0 [91.0, 207.0] |
| 25-30 | 6,669 | — | 4.10 [2.37, 6.62] | 7.60 [6.40, 9.10] | 4.20 [3.10, 5.50] | 142.0 [98.0, 203.0] |
| 30-35 | 7,698 | — | 2.98 [1.60, 4.96] | 7.90 [6.60, 9.60] | 4.10 [3.00, 5.40] | 149.0 [102.0, 217.0] |
| 35-40 | 4,945 | — | 1.67 [0.80, 3.12] | 8.30 [6.80, 10.70] | 4.00 [2.90, 5.40] | 160.0 [106.0, 249.0] |
| p-value |  |  | <0.001^a,b,c,d,e,f,g^ | <0.001^a,b,c,d,e,f,g^ | <0.001^b,c,d,e,f,g^ | <0.05^a,d,e,g^ |
| FSH groups | | | | | | |
| normal | 16,538 | 31 [28, 35] | 3.53 [2.00, 5.81] | — | 3.90 [2.90, 5.10] | 147.0 [101.0, 212.0] |
| pre-POI | 4,043 | 33 [30, 36] | 1.75 [0.82, 3.35] | — | 4.80 [3.60, 6.60] | 155.0 [104.0, 235.0] |
| POI | 300 | 35 [31, 38] | 0.37 [0.12, 1.38] | — | 8.15 [3.80, 11.70] | 221.0 [109.0, 3295.8] |
| POF | 262 | 36 [32, 38] | 0.06 [0.06, 0.12] | — | 34.20 [23.52, 45.27] | 100.0 [58.3, 188.8] |
| p-value |  | <0.001^a,b,c,d,e,g^ | <0.001^a,b,c,d,e,g^ |  | <0.001^a,b,c,d,e,g^ | <0.001^a,b,c,d,e,g^ |
| AMH groups | | | | | | |
| normal AMH | 17,766 | 31 [28, 34] | — | 7.60 [6.40, 9.10] | 4.10 [3.00, 5.40] | 146.0 [100.0, 209.0] |
| DOR | 1,845 | 35 [32, 37] | — | 9.10 [7.10, 11.80] | 3.60 [2.60, 5.00] | 175.0 [111.0, 279.0] |
| severe DOR | 1,532 | 36 [33, 38] | — | 13.80 [8.60, 26.42] | 4.90 [3.20, 9.70] | 167.0 [100.0, 287.3] |
| p-value |  | <0.001^a,b,c,g^ |  | <0.001^a,b,c,g^ | <0.001^a,b,c,g^ | <0.001^b,c,g^ |

Supplemental table 1 Demographic information

Data are expressed as the median [interquartile range].

a p<0.05 for the comparison between 18-25 and 25-30 in age groups, between normal and pre-POI in FSH groups, between normal AMH and DOR in AMH groups.

b p<0.05 for the comparison between 18-25 and 30-35 in age groups, between normal and POI in FSH groups, between normal AMH and severe DOR in AMH groups.

c p<0.05 for the comparison between 18-25 and 35-40 in age groups, between normal and POF in FSH groups, between DOR and severe DOR in AMH groups.

d p<0.05 for the comparison between 25-30 and 30-35 in age groups, between pre-POI and POI in FSH groups.

e p<0.05 for the comparison between 25-30 and 35-40 in age groups, between pre-POI and POF in FSH groups.

f P<0.05 for the comparison between 30-35 and 35-40 in age groups, between POI and POF in FSH groups.

g Kruskal-Wallis ANOVA test.


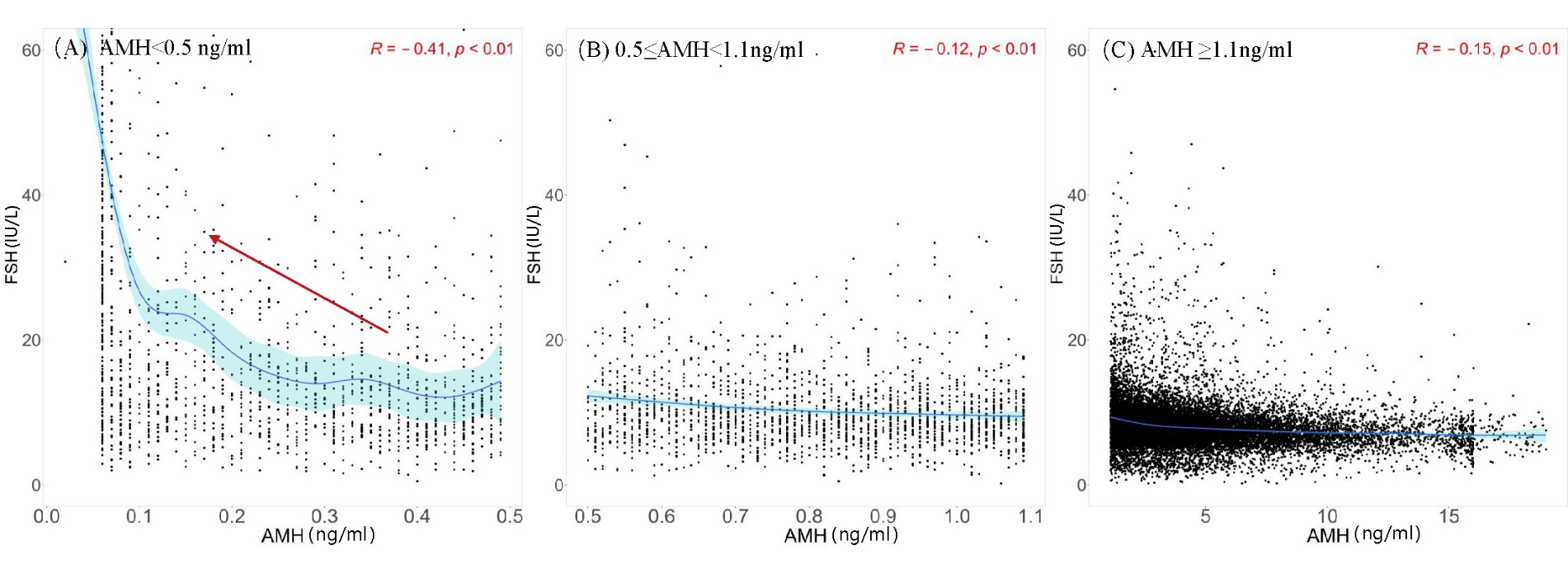


Supplemental figure 1. Scatterplots between AMH and FSH among different AMH groups. (A) AMH < 0.5 ng/ml; (B) 0.5 ≤ AMH< 1.1 ng/ml; (C) AMH ≥1.1ng/ml. The black dots illustrate the FSH levels of individuals across various ages. The blue solid line represents the trend line fitted through the data points, reflecting the overall trend of FSH levels with AMH. Light blue region represents the 95% confidence interval. Red arrow highlights the comparatively elevated FSH levels in individuals with lower AMH levels.


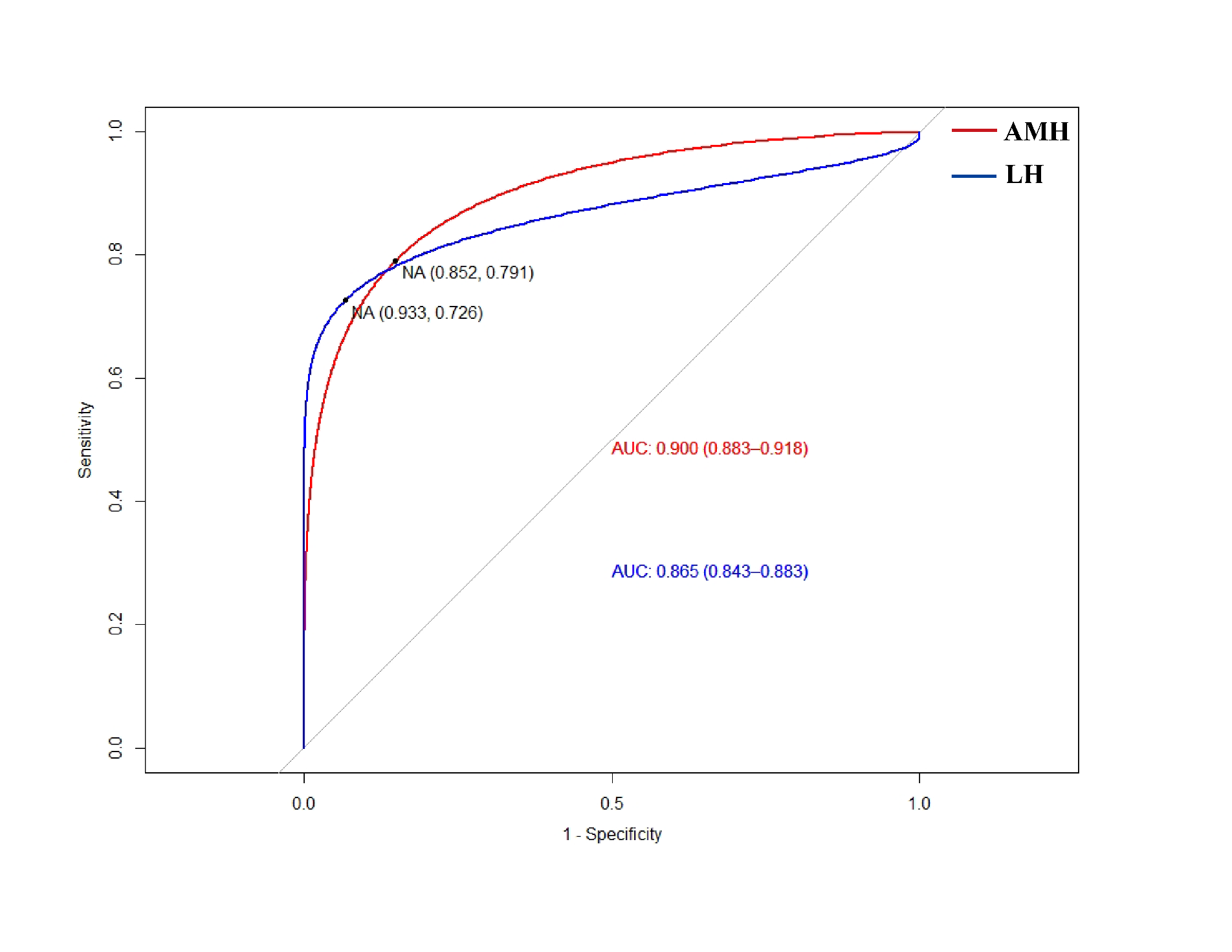


Supplemental figure 2 The ROC curves of AMH, LH for POI prediction. Red Line: AMH (Anti-Mullerian Hormone), AUC = 0.9; Blue Line: LH (Luteinizing Hormone), AUC = 0.865


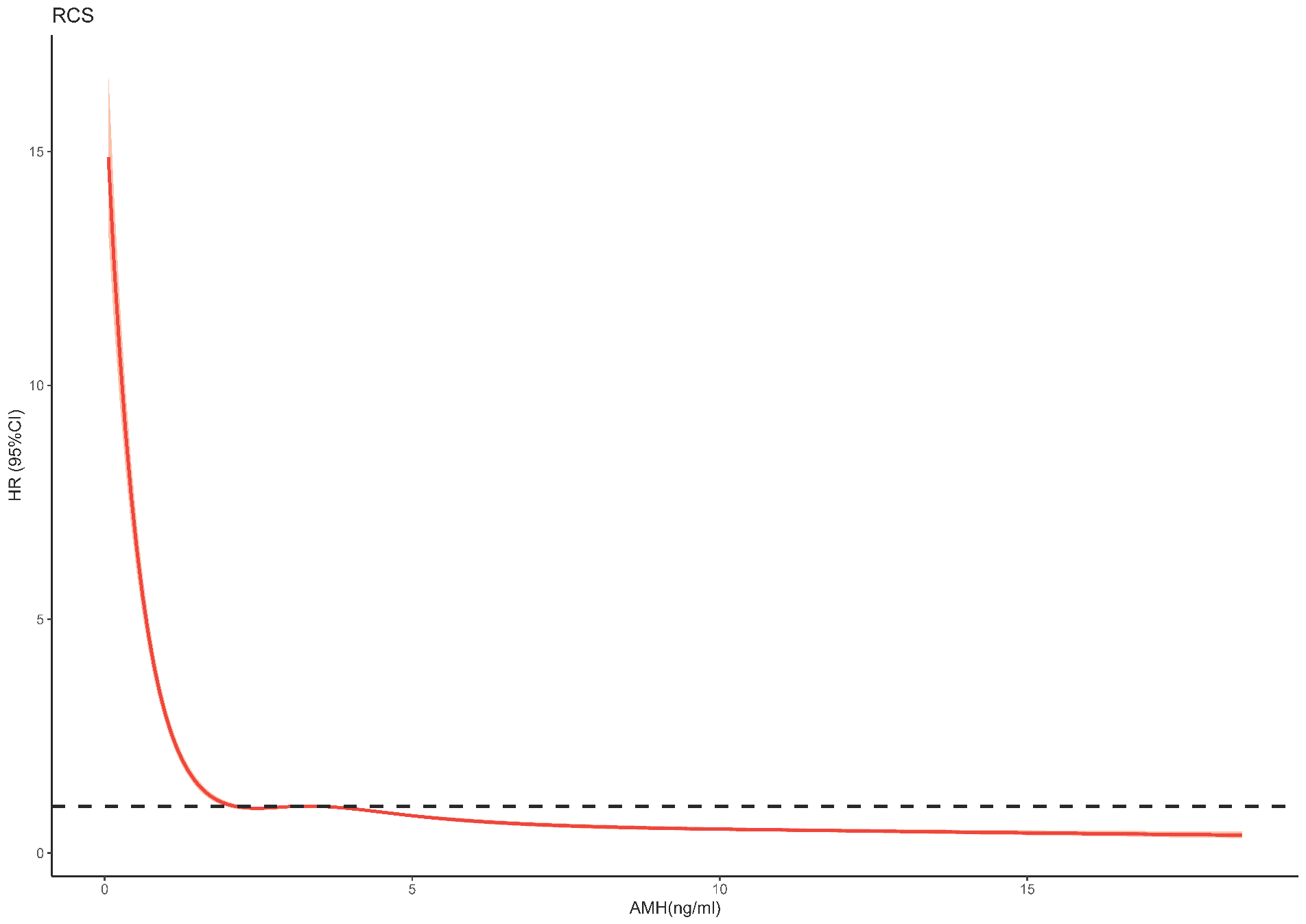


Supplemental figure 3 Age adjusted hazard ratios for POI according to levels of AMH on a continuous scale. Solid red lines are age adjusted hazard ratios, with dashed red lines showing 95% confidence intervals derived from restricted cubic spline regressions with seven knots. Reference lines for no association are indicated by solid bold lines at a hazard ratio of 1.0.


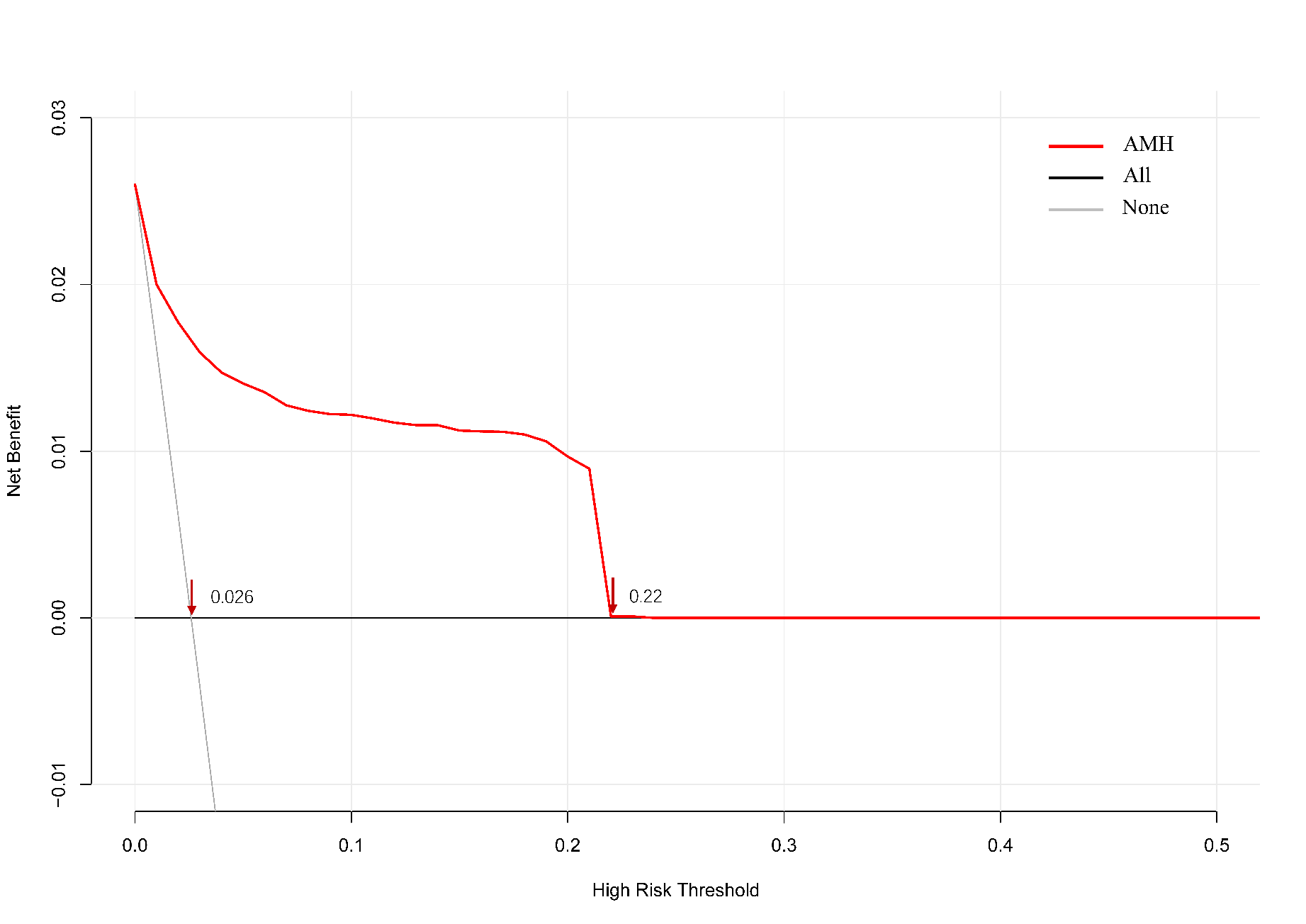


Supplemental figure 4 Decision curve analysis for the AMH. The y-axis measures the net benefit. The red line represents the AMH. Thick black line represents the assumption that patients use AMH to diagnose POI. Thin black line represents the assumption that all patients use AMH to diagnose POI. The net benefit was calculated by subtracting the proportion of all patients who are false positive from the proportion who are true positive, weighting by the relative harm of forgoing treatment compared with the negative consequences of an unnecessary treatment. Here, the relative harm was calculated by [Math Processing Error]. “[Math Processing Error]” (threshold probability) is where the expected benefit of treatment is equal to the expected benefit of avoiding treatment; at which time a patient will opt for treatment informs us of how a patient weighs the relative harms of false-positive results and false-negative results ([a − c]/[b − d] = [1 − pt]/pt); a − c is the harm from a false-negative result; b − d is the harm from a false-positive result. a, b, c and d give, respectively, the value of true positive, false positive, false negative, and true negative.
